# When it really counts: Investigating the relation between trait mindfulness and actual prosocial behavior

**DOI:** 10.1007/s12144-021-01860-y

**Published:** 2021-05-25

**Authors:** Simon Schindler, Stefan Pfattheicher

**Affiliations:** 1 Dresden University of Technology, 01069 Dresden, Germany; 2 Aarhus University, 8000 Aarhus C, Denmark

**Keywords:** Trait mindfulness, Prosocial behavior, Five facet mindfulness questionnaire

## Abstract

Meta-analytical findings suggested a positive link between trait mindfulness and prosociality. However, most correlational studies on mindfulness and prosociality have relied on self-report measures. The present work aimed to address this serious limitation by investigating actual prosocial *behavior*. We further focused on mindfulness as a multi-dimensional personality trait to disentangle effects of different mindfulness aspects. In addition, we tested whether the relation between trait mindfulness and prosocial behavior emerges under a theoretical meaningful experimental boundary condition (i.e., feelings of guilt). In two studies (using four different samples; *N* = 1240), we did not find support for a positive link between trait mindfulness and (a) charitable donation and (b) behavior in an incentivized economic game, respectively. Evidence for manipulated guilt-level as a moderator was inconclusive. Taken together, the findings point to a more complex role of trait mindfulness for prosocial behavior. Limitations and ideas for further research are discussed.

Mindfulness is currently a hot topic in psychological research as well as in many parts of the society. Besides investigating the positive effects of mindfulness interventions on mental and physical health, a growing but still substantially smaller body of psychological literature addresses interpersonal outcomes (Creswell, 2017), especially the question whether mindfulness benefits others. Exploring its prosocial nature, mindfulness has been investigated as an intervention and a personality trait; in the present research, we focus on the latter. Results of a recent meta-analysis showed a positive relation between trait mindfulness and prosocial outcomes supporting the prosocial nature of mindfulness (Donald et al., 2019). We argue, however, that there are some aspects that seriously limit the evidence for the claim that trait mindfulness positively relates to prosocial *behavior*. Primarily, most previous correlational studies relied on self-report measures. In the present study, we address this gap by testing the relation between trait mindfulness and incentivized prosocial behavior—and in addition give this relation an additional chance by testing it under a theoretical meaningful experimental boundary condition (i.e., feelings of guilt).

## Mindfulness

Mindfulness in commonly conceptualized as an open and nonjudgmental awareness of one’s present-moment experiences (Brown & Ryan, 2003; Kabat-Zinn, 1990). Specifically, it includes meta-cognitive awareness of one’s internal mental experiences and observing them as such and to watch them come and go. That is, one takes the position of a neutral, nonjudging observer of one’s own emotions and thoughts, without reflecting on their valence or their content in general. A mindful person is assumed to recognize each experience without ruminating about and identifying with it too much and without entangling oneself in additional thoughts and emotions by judging them (Bishop et al., 2004; Chambers et al., 2009; Karremans & Papies, 2017). This state of present-moment awareness can be described as the opposite of unintentional mind wandering (Killingsworth & Gilbert, 2010) or acting on “auto-pilot” (Bargh & Chartrand, 1999) and is supposed to have widespread effects on human functioning and behavior (e.g., Brown et al., 2007).

Mindfulness has been investigated as both an intervention and a personality trait. In light of the person-situation debate (Fleeson, 2004), both perspectives are important to understand the whole picture since they address different questions. In the present research, we applied a trait perspective to address whether the frequency of being in a mindful state (trait mindfulness) relate to prosocial outcomes. In contrast, an intervention perspective asks whether increasing the situational level of mindfulness (through a mindfulness intervention) promotes prosocial outcomes. Although both perspectives are related, they are conceptually distinct and have different theoretical implications (Bless & Burger, 2016). When reviewing evidence on mindfulness effects, it is therefore important to distinguish between evidence from intervention studies and evidence from correlational studies that include mindfulness as a trait (Van Dam et al., 2018). In fact, most empirical studies addressed mindfulness as a personality trait and examined its relationship to other constructs in correlational designs (Brown & Ryan, 2003; Baer et al., 2006). Therefore, testing the prosocial nature of mindfulness with correlational studies makes a valuable contribution to the literature—are those who experience mindfulness more often in their lives more likely to engage in prosocial behavior?

Several self-report measures have been developed to capture differences in trait mindfulness (for an overview, see Bergomi et al., 2013). Having its scientific roots in clinical psychology, research on mindfulness has so far focused almost entirely on its positive effect on individual well-being, mental and physical health, showing significant correlations with a variety of cognitive and affective indicators, such as lower levels of anxiety, stress and neuroticism as well as higher levels of positive affect, vitality and subjective well-being (e.g., Brown & Ryan, 2003; Baer et al., 2006). One major reason for the positive effects on well-being is that mindfulness is linked to more effective emotion regulation, self-regulatory capacity and executive control, and less emotional reactivity (e.g., Hill & Updegraff, 2012).

## Trait Mindfulness and Prosociality

Although mindfulness research in the interpersonal context is still in its infancy (Creswell, 2017), the literature so far suggests an almost unitary picture: mindfulness benefits others—children, romantic partners, friends, colleagues, or strangers (e.g., Geurtzen et al., 2015; Karremans et al., 2017). Surveying the mindfulness literature basically reveals two lines of arguments regarding why mindfulness is linked to prosociality: First, mindfulness is assumed to improve the ability of sustained attention and self-regulatory capacity (Chiesa et al., 2011). This further attenuates automatic and impulsive processes and increases awareness of others’ needs in the social environment (e.g., Condon, 2019). Accordingly, it is argued that mindfulness should promote prosociality, especially in circumstances that typically inhibit prosociality (Berry et al., 2020), such as the presence of bystanders (Condon et al., 2013). Second, mindfulness is assumed to facilitate disengaging from mental contents by meeting them with nonjudgmental acceptance. This attenuates self-referential thoughts and emotions, further reducing boundaries between self and others and in turn increasing empathic concern (Berry et al., 2018). According to these assumptions, both dimensions, acting with awareness and nonjudgmental acceptance, should be positively linked to prosocial behavior.

Donald et al. (2019) reported a positive significant correlation between trait mindfulness and prosocial outcomes across 12 studies including 32 effect sizes (*d* = .73, 95% CI [0.51, 0.96]); no evidence for publication bias was found. However, we argue that there are some aspects that seriously limit the evidence: First, most of the studies relied on self-reports (for the most recent example not included in this meta-analysis, see Guo et al., 2021). This is a serious limitation given that correlations between self-report measures can be inflated by shared method variance, especially when the measured constructs are socially desirable as in the present case. Only seven studies in the meta-analysis investigated actual prosocial behavior; *all of them* measured the extent to which the subject offered to help via email to an alleged (actually non-existent) victim of ostracism or by measuring the total number of ball throws towards an alleged (actually non-existent) victim in an online ostracism paradigm (Berry et al., 2018; Ridderinkhof et al., 2017). Second, the observed effect sizes for actual behavior were only small-to-medium (*d* = .37) in contrast to self-reports (*d* = .89). Third, 17 of the 32 effect sizes resulted from three surveys on self-reported parenting attitudes such as responsiveness (Geurtzen et al., 2015; Parent et al., 2016a, 2016b). Fourth, in none of the included studies did participants’ decisions involve any substantial costs for themselves when benefitting another person. In the included studies using actual behavior, all participants played cyberball and were then asked to write an e-mail to the other players while the degree of support towards the ostracized player served as the dependent variable. Given that trait mindfulness was unrelated to writing time in terms of total word count, writing a supportive e-mail as a requested task in the experiment did not entail substantial costs. This is a serious limitation concerning ecological validity, given that prosocial behavior often comes at a personal cost (Kawamura et al., 2020; Thielmann et al., 2020), for example, in terms of money (e.g., donations) or time (e.g., voluntary service). Moreover, extensive social psychology literature suggested the link between intention and behavior to be imperfect (e.g., Ajzen, 1991). Last, many consider psychology as science of behavior and should therefore not only rely on introspective self-reports and questionnaire ratings (Baumeister et al., 2007).

In the meta-analysis of Donald et al. (2019), most of the included studies used the one-dimensional Mindful Attention and Awareness Scale (Brown & Ryan, 2003), only two studies included measures of multiple facets of mindfulness such as the nonjudgmental acceptance dimension. These multiple aspects, however, have not been considered in the analysis by Donald et al. (2019), although both dimensions – awareness *and* nonjudgmental acceptance – can be seen as defining aspects in the classic conceptualization of mindfulness (Baer et al., 2006; Kabat-Zinn, 1990). Given the advantage of correlational studies to isolate effects and to investigate the specific role of single dimensions of a multi-dimensional trait such as mindfulness, it seems valuable to investigate the impact of acting with awareness and nonjudgmental acceptance on prosocial behavior separately.

### The Present Research

Studies addressing trait mindfulness and prosocial behavior so far suggest a positive correlation (Donald et al., 2019). However, these studies rarely assessed *actual* prosocial behavior and lack the involvement of personal costs (such as money or time). The present work aims to fill this relevant gap by investigating prosocial economic decisions that involve personal financial costs. Second, while Donald et al. (2019) analyzed trait mindfulness as a one-dimensional construct, we take a multi-dimensional trait perspective, in particular to disentangle the effects of the nonjudgmental and awareness facets, given that both dimensions are defining aspects in the mindfulness concept (Baer et al., 2006; Kabat-Zinn, 1990). In two studies, we tested the hypothesis that trait mindfulness is positively linked to actual prosocial behavior. In both studies, we included the Five Facet Mindfulness Questionnaire (FFMQ; Baer et al., 2006). In Study 1, we assessed donation behavior to a charitable organization. In Study 2, we assessed actual prosocial behavior in a dictator game. We chose the dictator game because (a) measures of individual differences (e.g., personality traits) were shown to be highly influential here (Thielmann et al., 2020), and (b) the dictator game is a parsimonious paradigm that models prosocial behavior in its simplest way: giving money to another individual.

At this point, we want to emphasize that the studies reported in the following and the failed evidence for a positive relation between trait mindfulness and prosocial behavior should not be considered as a final answer on the debate about the prosocial nature of mindfulness. Rather, the findings should be considered as an empirical argument for the idea that the relation between trait mindfulness and prosocial behavior is far more complex than initially thought. We discuss this issue in more depth in the General Discussion.

### Sample Characteristics and Statistical Power

Using G*Power (Faul et al., 2009), we conducted a power analysis for a two-tailed test to detect at least small to medium effects (ρ = .25) in our studies. Power was set to .90. This power analysis revealed a required sample size of *N* = 160 to detect a significant effect (alpha level of .05) given the existence of a true effect. However, as we did not know the size of the “true effect,” we oversampled when finances and participant accessibility made doing so feasible. Specifically, we preregistered data collection of 300 participants in Study 1, 500 participants in Study 2B, and 300 participants in Study 2C. We only fell short of this threshold in Study 2A. This was due to limited financial resources at that time, because the study was conducted in the lab and was much more expensive. Sample characteristics and descriptive values of prosocial behavior of all studies can be found in Table 1. Note, that Study 1 was conducted after the other studies.

**Table 1:** Overview of sample characteristics and descriptive values of prosocial behavior in Studies 1 and 2

Study	Country	Locale	*N*	*M*_age_	*SD*_age_	% women	Scale range	*M*	*SD*
Study 1	Germany	Online (Prolific)	306	29.4	9.3	43.3	binary	69.9^a^	–
Study 2A	Germany	Offline (lab)	109	22.8	4.4	45.0	0 to 4 euros	1.34	1.12
Study 2B	U.S.	Online (MTurk)	525	39.9	13.7	52.6	0 to 60 cents	16.86	15.46
Study 2C	Germany	Online (Prolific)	300	29.7	9.3	43.3	0 to 60 cents	14.83	14.73

MTurk = Amazon Mechanical Turk`

^a^Percentage of participants who donated their 50 cent bonus

### Research Ethics Statement and Data Handling

The studies reported in the present contribution were conducted in full accordance with the Ethical Guidelines of the German Association of Psychologists (DGPs) and the American Psychological Association (APA). Institutional review boards or committees are not mandatory at German universities (where the principal investigator of the studies was employed). In Studies 2A-C, we used deception (i.e., false performance feedback and alleged participation of other persons) when we experimentally manipulated feelings of guilt. Participants were debriefed at the end of the study. Data and the material of all studies, detailed results, preregistration protocols of Studies 1, 2B, and 2C, and additional preregistered exploratory analyses are available on the OSF (https://osf.io/wuzh2).

## Study 1: Donation

With this study, we wanted to test the general claim that trait mindfulness is positively linked to actual prosocial behavior by measuring donation behavior to a charitable organization. Sample characteristics can be found in Table 1.

### Method

#### Trait Mindfulness

The general tendency to being mindful in daily life was assessed by using the 39 items (α = .89) of a validated German version of the FFMQ (Michalak et al., 2016). The FFMQ contains five subscales: (1) nonjudging of experience (8 items, e.g., “I tell myself that I shouldn’t be feeling the way I’m feeling“; α = .93), (2) acting with awareness/automatic pilot/ concentration/nondistraction (8 items, e.g., “It is easy for me to concentrate on what I’m doing“; α = .84), (3) nonreactivity to inner experience (7 items, e.g., “I perceive my feelings and emotions without having to react to them“; α = .85), (4) overserving noticing/attending to sensations/perception/thoughts/feelings (8 items, e.g., “I notice how my emotions express themselves through my body“; α = .78), and (5) describing/labeling with words (8 items, e.g., “I’m good at finding the words to describe my feelings“; α = .91). The scales ranged from 1 (*never or very rarely true*) to 5 (*very often or always true*).

#### Prosocial Behavior

Participants were given a 50-cent bonus and told that they could keep the money for themselves or donate it to a charitable organization, the German Red Cross, to support their fight against Coronavirus (data was conducted at the beginning of the pandemic in March 2020). Participants then made their choice.

### Results

The overall score of the FFMQ was not significantly correlated with donation choice; this was also not the case for the five facets of the FFMQ (see Table 2). Furthermore, we conducted a multiple logistic regression analysis with all five facets of the FFMQ as predictors and donation choice as outcome variable. None of the five facets significantly predicted donation choice (see Table 3). Controlling for perceived fairness of participation payment (as preregistered as exploratory analysis) did not change the effects in terms of levels of significance.

**Table 2:** Zero-order correlations between the prosocial behavior, the overall FFMQ, and the five facets separately in Study 1 (*N* = 306), Study 2A (*N* = 109), Study 2B (*N* = 525), Study 2C (*N* = 300), and across Studies 2A-C in an internal meta-analysis (*N* = 934)

Variable	Study 1	Study 2A	Study 2B	Study 2C	Across Studies 2A-C
Overall FFMQ	0.07 [−0.05, 0.18]	0.07 [−0.12, 0.25]	**−0.19 [−0.27, −0.11]**	0.01 [−0.11, 0.12]	−0.05 [−0.21, 0.11]
Nonjudge facet	0.11 [−0.01, 0.22]	−0.08 [−0.26, 0.11]	**−0.34 [−0.41, −0.26]**	0.05 [−0.07, 0.16]	−0.13 [−0.38, 0.11]
Act aware facet	0.01 [−0.10, 0.12]	0.05 [−0.14, 0.23]	**−0.32 [−0.40, −0.24]**	−0.01 [−0.12, 0.11]	−0.11 [−0.34, 0.13]
Nonreact facet	0.07 [−0.05, 0.18]	0.09 [−0.10, 0.27]	**0.21 [0.12, 0.29]**	−0.01 [−0.12, 0.11]	0.10 [−0.04, 0.25]
Observe facet	−0.05 [−0.16, −0.06]	0.08 [−0.12, 0.26]	**0.15 [0.07, 0.24]**	0.03 [−0.08, 0.15]	**0.10 [0.01, 0.18]**
Describe facet	0.05 [−0.06, 0.16]	0.10 [−0.09, 0.28]	**−0.10 [−0.18, −0.01]**	−0.05 [−0.16, 0.06]	0.05 [−0.06, 0.15]

Bold marked correlations are significant with *p* < .05. Positive correlations indicate that prosocial behavior increases with stronger trait mindfulness. 95% confidence intervals are reported in brackets

**Table 3:** Summary of multiple regression analysis of the five facets of the FFMQ Predicting donation choice in Study 1 (*N* = 306)

	Parameter estimates					
	*B*	*SE*	*p*	*Exp(B)*	*LLCI*	*ULCI*
Nonjudge facet	0.22	0.16	.164	1.25	0.91	1.71
Act aware facet	−0.13	0.22	.547	0.88	0.58	1.34
Nonreact facet	0.16	0.21	.449	1.17	0.78	1.77
Observe facet	−0.16	0.21	.431	0.85	0.57	1.27
Describe facet	0.10	0.18	.587	1.10	0.78	1.56

Donation choice (0 = no donation, 1 = donation). CI refers to the 95% confidence interval

### Discussion

Results of this study revealed no support for the claim that trait mindfulness is positively linked to prosocial behavior. Neither the overall FFMQ, nor any of the five facets significantly predicted donation choice. A dichotomous measure of behavior (as used in this study) may have less potential to find common variance between the variables of interest than a continuous variable. Therefore, in the next study, we assessed prosocial behavior with a continuous variable.

## Study 2: Dictator Game

In this study, we investigated the positive link between trait mindfulness and prosociality by assessing behavior in a dictator game. We chose the dictator game because (a) measures of individual differences (e.g., personality traits) were shown to be highly influential here (Thielmann et al., 2020), and (b) the dictator game is a parsimonious paradigm that models prosocial behavior in its simplest way: giving money to another individual.

In addition, in this study, we experimentally manipulated guilt-level to explore whether the relationship between the *nonjudge facet* and prosocial behavior depends on the perceived levels of guilt (Bless & Burger, 2016). While prosocial behavior can be motivated by altruism (Batson & Powell, 2003), it often results from the egocentric goal of avoiding and reducing aversive states, such as feelings of guilt and distress (Cialdini et al., 1997; Dovidio et al., 1991; Graton & Ric, 2017; Penner et al., 2005). That is, the stronger people experience or anticipate feelings of guilt, the more likely they will engage in prosocial behavior. It can further be suggested that trait mindfulness and especially the nonjudgmental acceptance aspect are in general negatively related to feelings of stress and guilt because they are based on subjective negative judgments about a situation (Cameron & Fredrickson, 2015; Friese & Hofmann, 2016). If such judgments are reduced by a tendency to accept present-moment experiences without automatic judgment, aversive feelings (e.g., guilt) and their behavioral consequences are attenuated. Thus, by manipulating guilt-level, we addressed the idea that trait mindfulness and especially the nonjudgmental acceptance facet could even be *negatively* linked to prosocial behavior, because of a presumed negative relation between this facet and feelings of guilt. Overall, with the next study, we provide the relation between trait mindfulness and prosocial behavior an additional (theory-based) chance by investigating an additional boundary condition under which the relation might emerge more likely.

### Method

#### Procedure

In total, we collected data for three diverse studies (Studies 2A to 2C) from two countries, both online and in the lab. Sample characteristics can be found in Table 1. All three studies followed the same procedure: first, the assessment of trait mindfulness, followed by an experimental guilt manipulation, and finally, an assessment of prosocial behavior in a dictator game. Participants were randomly assigned to one of the guilt conditions (high guilt vs. low guilt).

#### Trait Mindfulness

In all three studies, participants completed the original or the validated German version of the FFMQ. In Study 2A, the scales ranged from 1 (*never or very rarely true*) to 7 (*very often or always true*). In Studies 2B and 2C, the scales ranged from 1 (*never or very rarely true*) to 5 (*very often or always true*). Reliability was good for the overall FFMQ and all subscales (all αs > .70, except for nonreactivity to inner experience in Sample 2A, α = .59).^1^

#### Guilt-Level Manipulation

To vary levels of guilt, we used the procedure of Schindler et al. (2019) where participants harmed another participant financially. Specifically, in the high guilt condition, participants were told that due to their performance on a task (identifying hidden faces in eight pictures), the financial outcome of another participant, “Person A,” was reduced. In the low guilt condition, participants learned that the financial outcome of another participant would not be reduced (Studies 2A and 2B) or increased (Study 2C).

After the task, participants’ outcome totaled 4 euros in Study 2A and 60 cents (Euro) in Studies 2B and 2C. In the high guilt conditions of all studies, the outcome of “Person A” totaled 0 euros. The outcome of “Person A” in the low guilt condition varied across studies: 2 euros in Study 2A, 30 cents in Study 2B, and 60 cents in Study 2C. The exact procedure can be found on the OSF.

#### Prosocial Behavior

To measure prosocial behavior, participants played a dictator game—that is, they were told that they now have the option to give a fraction of their outcome to “Person A.” They answered on a scale ranging from 0 to 4 euros (Study 2A) or 0 to 60 cents (Study 2B and 2C).

### Results

#### The Effect of Trait Mindfulness on Prosocial Behavior across Experimental Conditions

An overview about the results can be found in Tables 2 and 4. Significant correlations across the experimental conditions only emerged in Study 2B. Here, the overall score of the FFMQ was *negatively* correlated with prosocial behavior, *p* < .001; this was also the case for the nonjudge facet, the act aware facet, and the describe facet *p*s < .001 (see Table 2). Positive significant correlations emerged with nonreact facet and the observe facet.

**Table 4:** Multiple linear regression results of participants’ amount of given money in the dictator game as a function of the FFMQ facets, the guilt-level manipulation, and the interaction effect between the Nonjudge facet and the guilt-level manipulation in Study 2A (*N* = 109), Study 2B (525), and Study 2C (*N* = 300)

		Parameter estimates								
		Study 2A			Study 2B			Study 2C		
Step	Predictors	*B*	*SE*	*p*	*B*	*SE*	*p*	*B*	*SE*	*p*
(1)	Nonjudge facet	−0.15	0.12	.213	**−3.52**	**1.02**	**.001**	1.38	1.02	.178
	Act aware facet	0.04	0.12	.720	−2.07	1.08	.055	−0.24	0.98	.810
	Nonreact facet	0.12	0.12	.289	**3.16**	**1.13**	**.005**	−0.39	0.95	.684
	Observe facet	0.05	0.11	.641	0.08	1.21	.947	0.90	0.90	.315
	Describe facet	0.10	0.11	.394	0.27	1.28	.833	−1.07	0.94	.260
(2)	Guilt	**0.25**	**0.11**	**.027**	0.93	0.63	.139	**3.34**	**0.84**	**< .001**
(3)	Guilt × Nonjudge facet	**−0.25**	**0.11**	**.020**	1.19	0.63	.058	−1.15	0.85	.856

The FFMQ facets were z-standardized; low guilt-level = −1, high guilt-level = 1. Significant values are marked in bold face

In a multiple regression analysis across the experimental conditions including all five subscales, the nonjudge facet and the nonreact facet were significant predictors in Study 2B (see Table 4). Here, the nonjudge facet was the strongest predictor and was negatively related to prosocial behavior whereas the relationship with the nonreact facet was positive. In Studies 2A and 2C, none of the facets was a significant predictor.

To gain more precise estimates, we performed meta-analyses across the three studies (*N* = 934) on the zero order correlations between the overall FFMQ as well as the five facets and prosocial behavior (see Table 2). There was a significant, positive but small correlation between prosocial behavior and the observe facet. All other correlations were not significant.

#### Nonjudge Facet × Guilt-Level Interaction

In Study 2A, a significant guilt level × nonjudge facet interaction emerged (see Table 4). Simple slope analyses revealed that the nonjudge facet significantly predicted the given amount of money in the high guilt condition, *B = −*0.32, *SE =* 0.16, *p =* .047, 95% CI [−0.64, −0.00], but not in the low guilt condition, *B =* 0.18, *SE =* 0.16, *p =* .259, 95% CI [−0.14, 0.51]. Of note, the relation between the nonjudge facet and the given amount of money emerged to be *negative* in the high guilt condition. In Study 2B, the interaction was not significant, but it approached the level of *p* = .05; however, the pattern contradicted the results from Study 2A, showing a *positive* relation between the nonjudge facet and the given amount of money in the high guilt condition. No significant interaction effect between the nonjudge facet and guilt-level occurred in Study 2C. To provide an integrative data analysis for the interaction effect across Studies 2A-C, we *z*-standardized the five facets as well as the dependent measure and merged the three data sets into one single data set. Results of a linear regression analysis revealed no significant interaction effect, *B =* 0.23, *SE =* 0.03, *p =* .464, 95% CI [−0.04, 0.09].

### Discussion

Overall, Study 2 revealed no clear support for a positive relationship between trait mindfulness and prosocial behavior. Significant zero-order correlations across the experimental conditions only occurred in Study 2B, but the correlation between the overall FFMQ and prosocial behavior was *negative*. Further, three of the five significant correlations between prosocial behavior and the five facets were also negative, among them the two most important facets (nonjudge and acting aware). Across all three studies, only the correlation between prosocial behavior and the observe facet was significant, but small.

There was a significant interaction between the nonjudge facet and guilt-level in Study 2A, indicating that the nonjudge facet was *negatively* linked to prosocial behavior under conditions of high state guilt, but not in the low guilt condition. We want to acknowledge, however, that Study 2A only had low statistical power. Given that the interaction was almost significant with the opposite pattern in Study 2B and not significant in Study 2C, results remain inconclusive. In light of the valid theoretical arguments for the role of guilt-level, this idea deserves further research.

## General Discussion

In the present work, predictability of trait mindfulness was tested for actual prosocial behavior. Findings of two studies did not reveal support for a positive relation. In contrast to these findings, in a recent meta-analysis, Donald et al. (2019) overall obtained a significant positive relationship between trait mindfulness and prosocial outcomes. However, the included studies were mostly based on *reported* prosocial behavior and half referred to self-reported parenting attitudes. The included studies investigating actual behavior lack ecological validity in the sense that prosocial behavior often involves costs for individuals when benefitting another person. Regarding the present findings, the conclusion of a general positive relationship between trait mindfulness and *actual* prosocial behavior appears to be premature and points to so far neglected boundary conditions.

Contradicting the existing literature on mindfulness, we addressed the idea that trait mindfulness and especially the nonjudgmental acceptance facet could even be negatively linked to prosocial behavior. Given that one important aspect of mindfulness refers to nonjudgmental acceptance of present experiences, aversive feelings such as guilt should be attenuated. Assuming that people often engage in prosocial behavior to avoid or to reduce feelings of guilt (Graton & Ric, 2017; Penner et al., 2005), attenuating these feelings would lead to *less* prosocial behavior. Thus, as a potential moderator for the relation between trait mindfulness and prosocial behavior, we manipulated feelings of guilt when having the possibility to engage in prosocial behavior. Despite the vast number of published articles on mindfulness, we are unaware of any empirical work linking the nonjudgmental aspect of mindfulness to actual prosocial behavior. In our studies, the overall evidence for manipulated guilt-level as a moderator was inconclusive: In Study 2A, prosocial behavior and the nonjudge facet were significantly negatively correlated in the high but not the low guilt condition. Study 2B revealed evidence in the opposite direction (a positive correlation in the high- but not in the low-guilt condition). In Study 2C, no moderation by guilt level was found. It could be that participants did not fully believe our coverstory about their performance feedback and the existence of “Person A” — especially in Studies 2B and 2C which were conducted online. However, we did not assess study suspicion or general thoughts about the paradigm, so this is speculative. Moreover, using one-shot financial decisions to test this idea might not be the best choice to test this idea (Fleeson, 2004; see limitations below); a repeated measure of prosocial behavior would have been a better choice. Given the plausible theoretical arguments for the role of guilt level, this idea deserves further research using improved methods.

We want to acknowledge several limitations of the presented work: First, prosocial behavior in the present studies is limited to financial behavior. Although this kind of behavior is part of everyday life, future research should thus investigate prosocial behavior in different areas (e.g., blood donation, engaging in voluntary activities). It may be that dispositional mindfulness is more strongly related to prosocial behavior in personal interactions (where compassion is more present) rather than in economic games. Furthermore, economic games reflect rather momentary behaviors rather than typical individual’s behavior and thus might be more prone to situational variables than traits (Fleeson, 2004). Nonetheless, the present studies reflect informative evidence for the field by showing that the relation between trait mindfulness and prosocial behavior is not as straightforward as it is described in the existing literature. In this regard, our study makes an important contribution and points to a more complex pattern of mindfulness and prosociality that needs further investigation.

Second, the correlative nature of our data does not allow us to draw any causal conclusions. Here, research on mindfulness interventions is more informative. Notably, the terms mindfulness and mediation are often used interchangeably in both public discourse and in scientific literature. Valid interpretation of empirical results must take a proper account of exactly what types of mindfulness and mediation interventions are involved (Schindler, 2020; Van Dam et al., 2018). Recent meta-analyses on the effects of various kinds of long-term meditation interventions (such as loving kindness meditation or mindfulness-based programs) on prosocial outcomes revealed mixed evidence: While Luberto et al. (2018) concluded that “meditation can improve prosocial emotions and behaviors” (p. 708), Kreplin et al. (2018) identified “a number of biases and theoretical problems that need addressing to improve quality of research in this area”. Exclusively meta-analyzing studies using experimental mindfulness interventions and assessments of actual prosocial behavior, Berry et al. (2020) reported an overall positive significant effect size (*Hedges’ g* = .43). However, in contrast to compassion-related outcomes, effect sizes referring to generosity (studies using economic games) or instrumental helping behavior outcomes clustered around zero and “may not be mutable to training in mindfulness” (Berry et al., 2020, p. 17). This observation speaks to the idea that prosocial effects of mindfulness might depend on the nature of prosocial behavior and might not hold for money-related helping behavior, rather for outcomes that are directly compassion-related.

Third, in the present studies, we exclusively relied on the FFMQ of Baer et al. (2006). This is an established scale measuring mindfulness as a multi-dimensional personality trait and – in contrast to single dimension scales – enabled us to disentangle effects of different mindfulness aspects. However, given the ongoing discussion about problems with measuring dispositional mindfulness (e.g., Choi et al., 2020), further assessments should be used in future research.

Taken together, the present research does not add to the absolute positive reputation of mindfulness in the interpersonal context. Instead, the findings point to a more complicated role of trait mindfulness for prosocial behavior.

## Data Availability

Data and the material of all studies, detailed results, preregistration protocols of Studies 1, 2B, and 2C, and additional preregistered exploratory analyses are available on the OSF (https://osf.io/wuzh2).

## Footnotes

1 In Study 2A, due to miscommunication, we missed including 14 of the 39 items. All subscales were assessed with 5 items.
